# Analyzing HIV Pre-exposure Prophylaxis and Viral Suppression Disparities: Insights From America’s HIV Epidemic Analysis Dashboard (AHEAD) National Database

**DOI:** 10.7759/cureus.67727

**Published:** 2024-08-25

**Authors:** Chiemelie C Oddie-Okeke, Oluwatoyin Ayo-Farai, Charity Iheagwara, Olayinka O Bolaji, Oluwatosin B Iyun, Shakhnoza Zaynieva, Okelue E Okobi

**Affiliations:** 1 Internal Medicine, Milton Keynes University Hospital, Milton Keynes, GBR; 2 Public Health, Jiann-Ping Hsu College of Public Health, Georgia Southern University, Statesboro, USA; 3 Infectious Diseases, Saint Michael's Medical Center, Newark, USA; 4 Cardiology, Midland Health Clinic, Midland, USA; 5 Public Health and Family Medicine, University of Cape Town, Cape Town, ZAF; 6 Medicine, Jefferson Torresdale Hospital, Philadelphia, USA; 7 Family Medicine, Larkin Community Hospital Palm Springs Campus, Hialeah, USA

**Keywords:** pre-exposure prophylaxis (prep), public health, ethnicity, race, gender, age, disparities, ahead national database, viral suppression, hiv

## Abstract

Despite advancements in human immunodeficiency virus (HIV) treatment and prevention, disparities in pre-exposure prophylaxis (PrEP) uptake and viral suppression persist across different demographics. This study analyzes data from America’s HIV Epidemic Analysis Dashboard (AHEAD) National Database to identify and understand these disparities based on age, gender, and race/ethnicity. In this study, we utilized the AHEAD National Database, which tracks HIV indicators across various demographics, including age, gender, and race/ethnicity. Data from 2017 to 2022 were analyzed to assess trends in PrEP uptake and viral suppression rates. Viral suppression was defined as having less than 200 copies of HIV per milliliter of blood. Data analyses were conducted to identify disparities and trends over time. The study has found notable disparities in PrEP uptake and viral suppression. From 2017 to 2022, PrEP prescriptions significantly increased from 13.20% to 36% of those eligible, rising from 161,185 to 437,425. During the same period, viral suppression rates among people with HIV rose from 63.10% to 65.10%, with the total number of individuals achieving viral suppression growing from 538,414 to 663,121. Younger individuals and males had higher uptake rates compared to females. Racial and ethnic disparities were also evident, with higher PrEP uptake and viral suppression rates among White and multiracial individuals compared to Black/African American and Hispanic/Latino populations. Viral suppression rates generally improved across all groups but remained lower for marginalized communities. In conclusion, while there has been overall progress in PrEP uptake and viral suppression, significant disparities persist. Targeted interventions are needed to address these gaps, particularly among marginalized racial and ethnic groups and underserved age demographics. Continued monitoring and tailored public health strategies are essential for achieving equitable HIV care and prevention.

## Introduction

Human immunodeficiency virus (HIV) remain a major public health challenge in the United States despite significant advancements in treatment and prevention. Approximately 1.2 million people in the United States are living with HIV [1]. The annual incidence of new infections has gradually declined, mainly due to improved treatment and prevention strategies, including the widespread use of pre-exposure prophylaxis (PrEP) [2]. The burden of HIV is evident in substantial healthcare costs and the impact on quality of life, with lifetime medical expenses for an individual with HIV estimated to exceed $300,000 [3]. The stigma associated with HIV can lead to social isolation and mental health issues, further exacerbating the burden [4,5].

PrEP has been transformative in HIV prevention, offering a highly effective method for reducing the risk of HIV acquisition among high-risk populations such as men who have sex with men and American African racial groups [6-8]. PrEP involves the daily use of antiretroviral medications by individuals who are HIV-negative but at high risk of infection [6]. By maintaining undetectable levels of HIV in the blood, PrEP is a crucial tool in the strategy to end the HIV epidemic. However, the effectiveness of PrEP and the rates of achieving viral suppression, defined as maintaining an HIV viral load of less than 200 copies per milliliter, vary across different demographic groups, reflecting disparities in healthcare access and outcomes. Achieving viral suppression improves health outcomes and reduces the likelihood of HIV transmission [6,7].

Despite effective treatments and prevention methods, significant disparities in PrEP utilization and viral suppression rates persist across age, gender, race, and ethnicity [8]. These disparities are influenced by factors such as socioeconomic status, access to healthcare, and cultural barriers [8]. For instance, men who have sex with men are more likely to use PrEP compared to other risk groups. Racial and ethnic minorities often experience lower rates of PrEP utilization and viral suppression, attributed to systemic inequalities in healthcare access and resources [7-9]. Age-related disparities also impact PrEP and viral suppression outcomes, with younger individuals facing unique challenges and older adults encountering different barriers. Understanding these differences is crucial for developing targeted interventions [9].

The Ending the HIV Epidemic (EHE) initiative aims to reduce new HIV infections by 75% by 2025 and 90% by 2030, focusing on improving access to prevention and treatment, enhancing data collection, and addressing health disparities [5]. America’s HIV Epidemic Analysis Dashboard (AHEAD) is a vital resource for addressing these disparities. Developed to support the EHE initiative and align with the national HIV strategy, AHEAD provides comprehensive data on HIV prevention, care, and treatment indicators, including PrEP utilization and viral suppression rates [10]. This study leverages AHEAD's data to analyze disparities in PrEP uptake and viral suppression across various demographic factors, including age, gender, race, and ethnicity.

## Materials and methods

Study design and population

This cross-sectional analysis examines data spanning from 2017 to 2022. The study focuses on disparities in PrEP uptake and viral suppression among different age groups, genders, and racial/ethnic groups. The population of interest includes individuals diagnosed with HIV who are either on PrEP or achieving viral suppression, with fewer than 200 copies of HIV per milliliter of blood.

Data source and description

This study analyzes disparities in HIV PrEP and viral suppression using data from the AHEAD. It is a comprehensive tool designed to support and track progress toward the EHE initiative in the United States and aligns with the national HIV/AIDS strategy. It provides a robust platform for monitoring key indicators of HIV care and prevention across various demographics [10].

Data extraction, variables, and data analysis

PrEP Uptake

The percentage of individuals prescribed PrEP relative to those with indications for PrEP was analyzed across different demographics.

Viral Suppression

It is defined as the percentage of people living with diagnosed HIV infection in a given year who have an amount of HIV less than 200 copies per milliliter of blood.

Demographic Variables

These include age group (13-24, 25-34, 35-44, 45-54, 55-64, 65+), gender (male, female, transgender), and race/ethnicity (American Indian/Alaska Native, Asian, Black/African American, Hispanic/Latino, Native Hawaiian/Other Pacific Islander, White, multiracial).

Quantitative Analysis

Quantitative analysis was performed to assess trends over time and identify disparities. Descriptive statistics were used to summarize PrEP utilization and viral suppression rates across different demographic groups. One-way analysis of variance (ANOVA) was conducted with the objective of evaluating the differences between groups. Trend analysis assessed changes over the years, focusing on improving or declining PrEP uptake and viral suppression rates. For this study, data extraction was performed using Statistical Analysis System (SAS) 9.4 software (SAS Institute Inc., Cary, United States), even as the data analysis was carried out using SAS 9.4 and R v.3.5.0 software (R Foundation for Statistical Computing, Vienna, Austria).

Ethical considerations

This data was extracted from publicly available primary data. Ethical approval is not required for publicly available data. All data from the source were anonymized, and no direct patient identifiers were accessed or reported.

## Results

Trends and disparities in HIV PrEP uptake

The analysis of disparities in HIV PrEP and viral suppression reveals significant trends and variations presented in Table 1. Between 2017 and 2022, the estimated percentage of individuals prescribed PrEP, relative to those with PrEP indications, increased substantially from 13.20% (n=161,185) to 36% (n=437,425). Despite these gains, the current PrEP uptake remains well below the 50% goals set for 2025 and 2030. Moreover, gender disparities remain prominent, with PrEP prescription rates for males rising from 15.10% in 2017 to 40.90% in 2022, compared to an increase for females from 5% (n=8059) to 14.40% (n=62,989). This disparity highlights a continued need to address gender-based differences in PrEP uptake. Age-related differences are also notable. Individuals aged 13-24 saw an increase in PrEP prescriptions from 7.70% (n=12,411) to 23.50% (n=102,795), while those aged 25-34 and 35-44 exhibited more substantial increases, reaching 39.60% (n=173,220) and 45.60% (n=199,466), respectively, by 2022. Based on the above data, one may derive important information regarding the age-related trends in PrEP uptake and the public health implications of trends. For instance, the data analysis has indicated a clear and comprehensible trend of increasing use of PrEP across different age groups, with individuals aged 24-44 years leading in the proportion of PrEP users. Nevertheless, the increase in PrEP use among younger persons aged 13-24 years, despite being significant, is still lagging behind the PrEP use rates observed in older cohorts. The public health implication of this finding is that the observed trends indicate that despite the increased use and awareness of PrEP in younger populations, there is still a need for more targeted interventions to enhance uptake in the population cohorts, owing to the elevated risk of HIV infection in younger populations.

**Table 1:** National data for PrEP coverage based on sociodemographic variables PrEP: Pre-exposure prophylaxis; -: Not available

National data for PrEP coverage	2017	2018	2019	2020	2021	2022
Estimated number of people prescribed PrEP	161,185	221,026	275,794	301,324	366,359	437,425
Percentage	13.20%	18.20%	22.70%	24.80%	30.10%	36%
National, Male (sex)	24,339 (15.10%)	45,752 (20.70%)	70,879 (25.70%)	84,672 (28.10%)	125,295 (34.20%)	178,907 (40.90%)
National, Female (sex)	8059 (5%)	15,472 (7%)	25,649 (9.30%)	31,338 (10.40%)	45,429 (12.40%)	62,989 (14.40%)
National, 13-24	12,411 (7.70%)	26,744 (12.10%)	41,369 (15%)	47,007 (15.60%)	71,440 (19.50%)	102,795 (23.50%)
National, 25-34	24,500 (15.20)	45,752 (20.70%)	71,706 (26%)	82,864 (27.50%)	123,829 (33.80%)	173,220 (39.60%)
National, 35-44	26,273 (16.30%)	47,521 (21.50%)	73,913 (26.80%)	91,301 (30.30%)	136,286 (37.20%)	199,466 (45.60%)
National, 45-54	22,566 (14%)	40,448 (18.30%)	59,572 (21.60%)	71,112 (23.60%)	97,451 (26.60%)	134,727 (30.80%)
National, 55-64	-	-	-	-	-	-
National, 65+	-	-	-	-	-	-
National, American Indian/Alaska Native	-	-	-	-	-	-
National, Asian	-	-	-	-	-	-
National, Black/African American	6,931 (4.30%)	-	-	-	-	-
National, Hispanic/Latino	12,411 (7.70%)	-	-	-	-	-
National, Native Hawaiian/Other Pacific Islander	-	-	-	-	-	-
National, White	57,221 (35.50%)	-	-	-	-	-
National, Multiracial	-	-	-	-	-	-

Racial and ethnic disparities show varying trends. Data for Black/African American and Hispanic/Latino populations was reported only in 2017, with 4.30% (n=6,931) and 7.70% (n=12,411) of these groups prescribed PrEP, respectively. For White individuals, PrEP uptake was notably higher at 35.50% (n=57,221). However, data for other racial and ethnic groups, including Asian, Native Hawaiian/Other Pacific Islander, and American Indian/Alaska Native, was not reported, indicating a gap in data collection and analysis. Overall, the data underscores progress in PrEP uptake but also highlights persistent disparities by gender, age, and race/ethnicity that need continued attention to ensure equitable access and adherence across all demographics.

Trends in viral suppression rates among people with HIV

From 2017 to 2022, the percentage of people with HIV achieving viral suppression increased from 63.10% to 65.10%, and the total number of individuals with viral suppression grew from 538,414 to 663,121. Despite these gains, the current suppression rate remains well below the 95% goals for 2025 and 2030. Continued efforts are needed to reach these targets and ensure broader, more effective treatment access and adherence.

Gender-based trends in viral suppression rates among people with HIV

**Figure 1:**
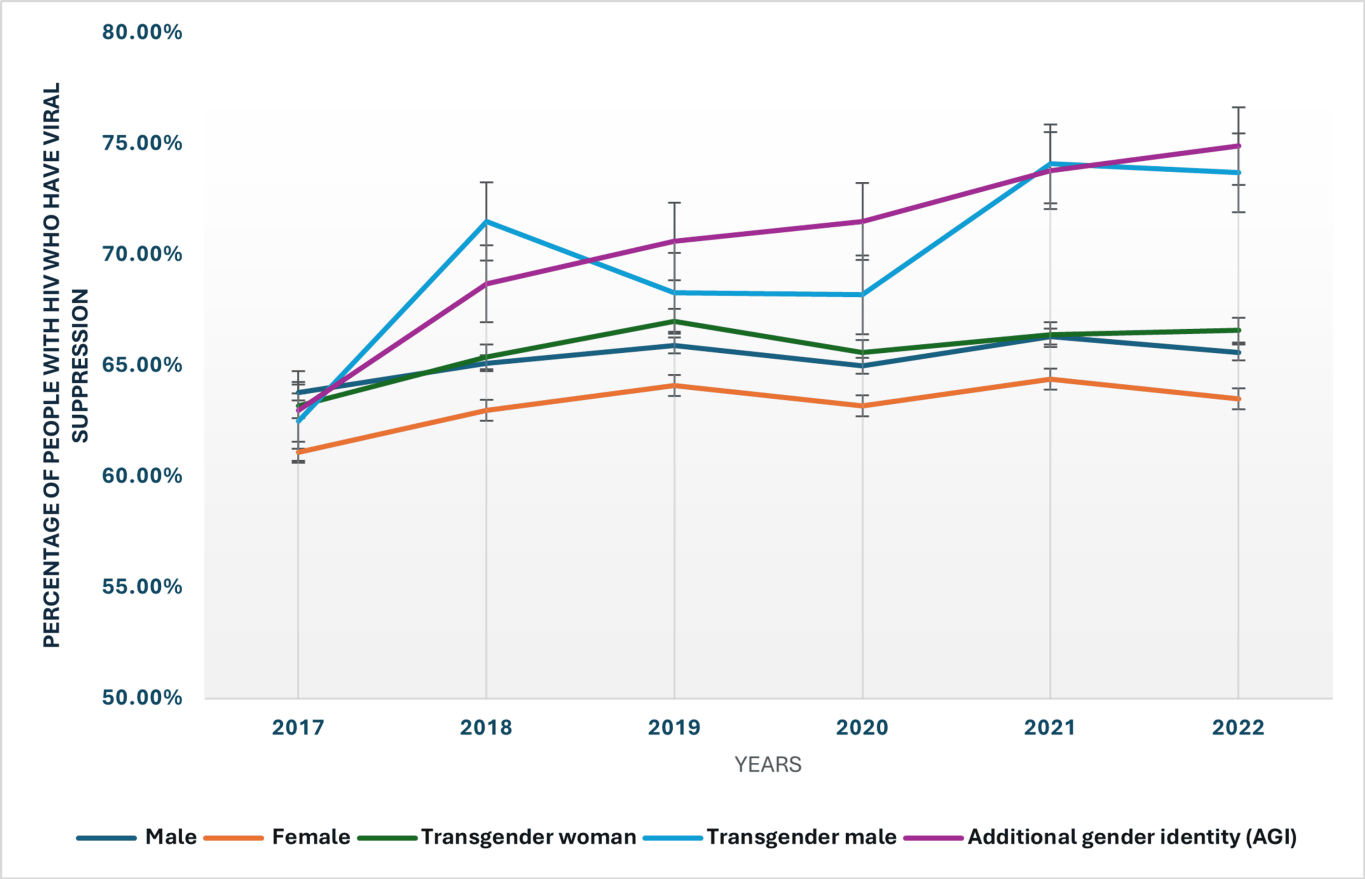
Viral suppression rates based on gender HIV: Human immunodeficiency virus

From 2017 to 2022, the percentage of individuals with HIV achieving viral suppression showed notable trends across different demographics (Figure 1). The viral suppression rate for males increased slightly from 63.80% in 2017 to 65.60% in 2022. Females experienced a similar trend, increasing from 61.10% to 63.50% over the same period. Transgender women showed a consistent improvement in viral suppression rates, rising from 63.20% in 2017 to 66.60% in 2022. Transgender males also exhibited a significant increase, with rates climbing from 62.50% to 73.70%. Additionally, individuals with additional gender identities (AGI) had the highest rate of viral suppression, increasing from 63% in 2017 to 74.90% in 2022. The trends indicate overall progress in viral suppression among all groups, though disparities persist.

Age-based trends in viral suppression rates among people with HIV

**Figure 2:**
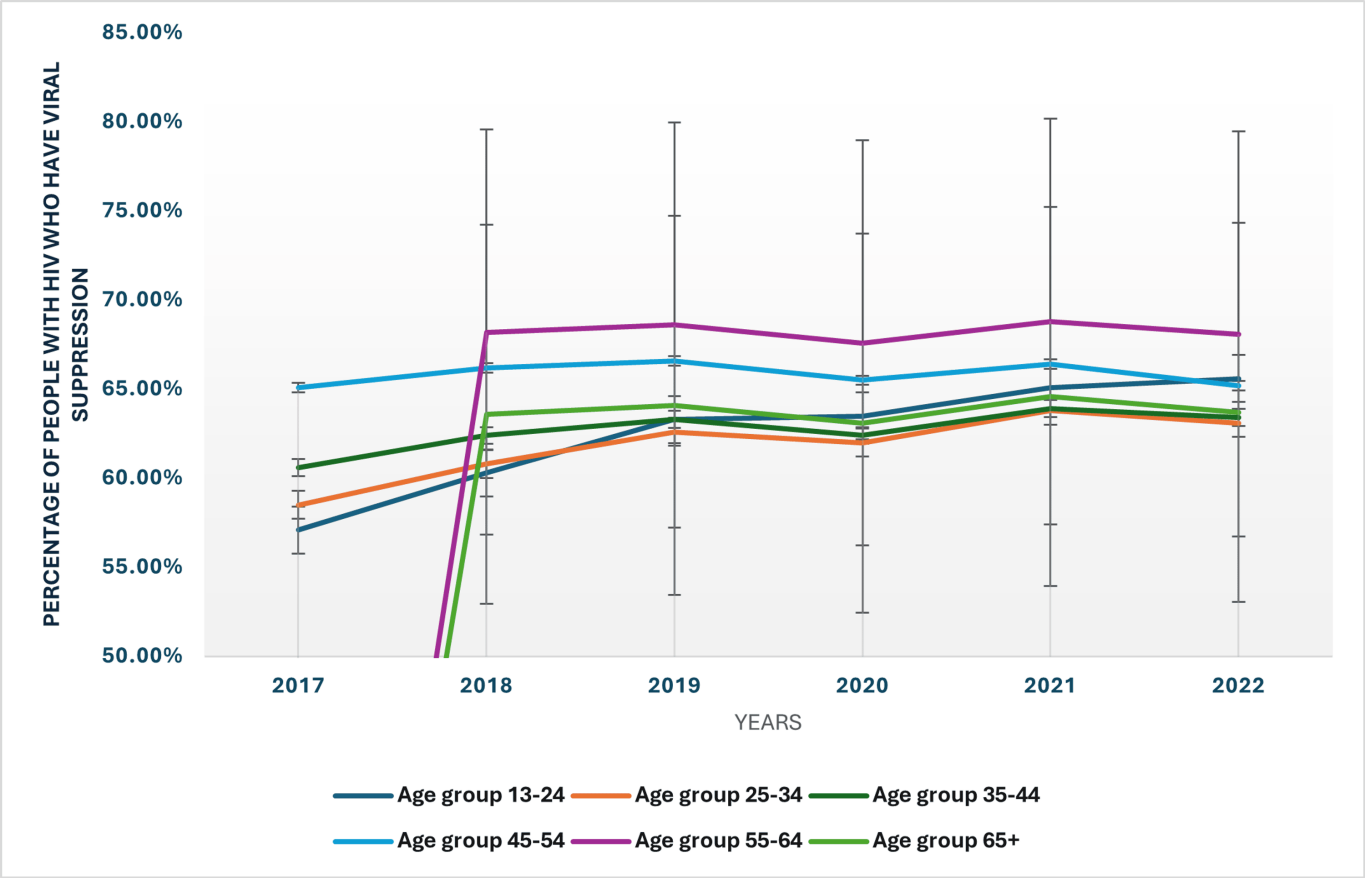
Viral suppression rates based on age groups HIV: Human immunodeficiency virus

From 2017 to 2022, the percentage of people with HIV achieving viral suppression varied across age groups (Figure 2). The youngest age group (13-24 years) increased from 57.10% in 2017 to 65.60% in 2022. Individuals aged 25-34 showed a more modest rise from 58.50% to 63.10% over the same period. Viral suppression rates for those aged 35-44 increased from 60.60% to 63.40%. The 45-54 age group exhibited a higher rate, starting at 65.10% and slightly decreasing to 65.20% by 2022. Data for individuals aged 55-64 became available in 2018, showing an increase from 68.20% in 2018 to 68.10% in 2022, with a slight fluctuation in between. The oldest group (65+) had a variable but overall steady suppression rate, beginning at 63.60% in 2018 and ending at 63.70% in 2022. These trends reflect overall improvements in viral suppression across all age groups, with some fluctuations indicating areas for targeted intervention.

Mode of HIV transmission trends in viral suppression rates

**Figure 3:**
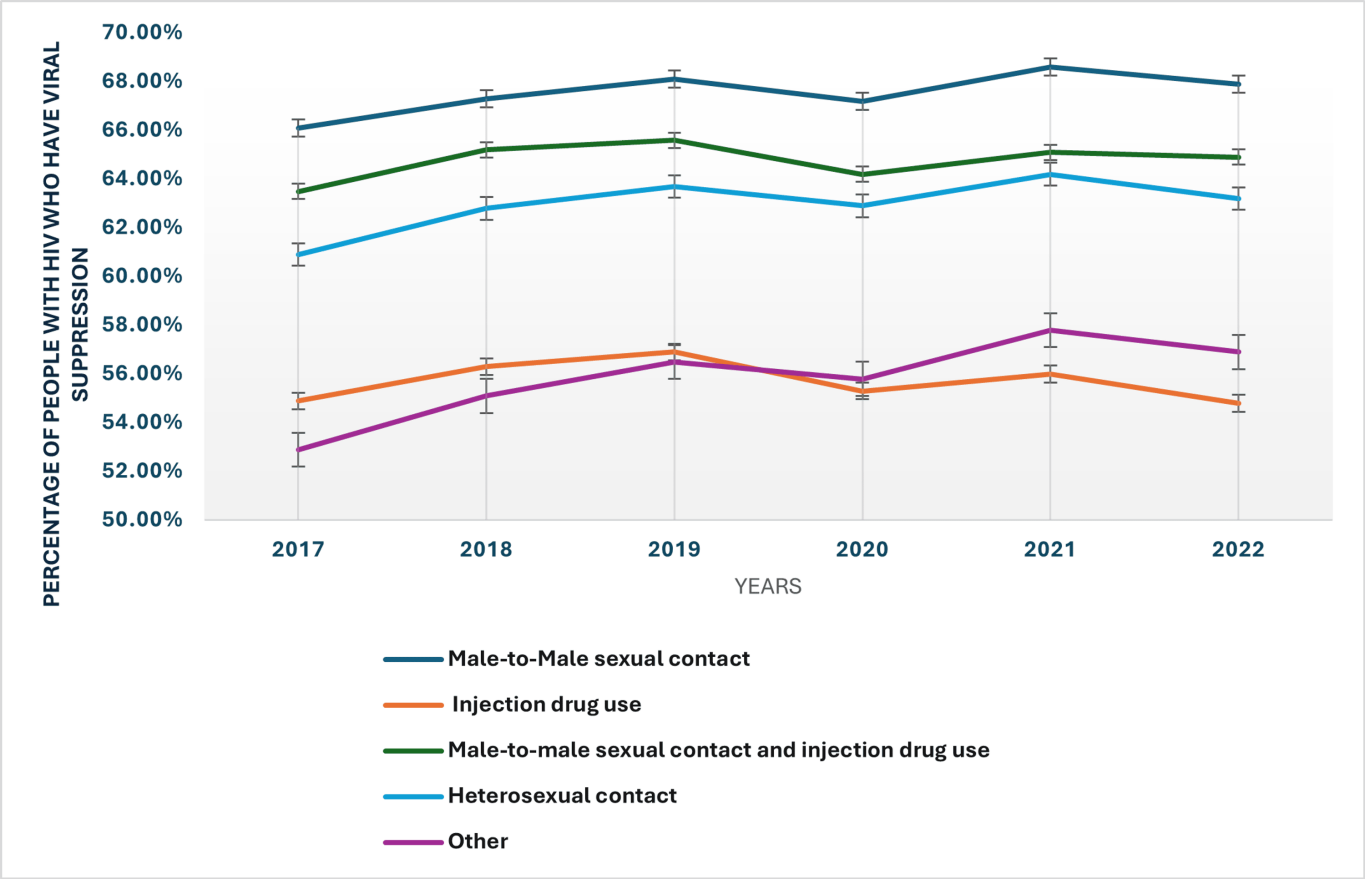
Mode of transmission in viral suppression rates HIV: Human immunodeficiency virus

From 2017 to 2022, the percentage of people with HIV achieving viral suppression varied by mode of transmission (Figure 3). Individuals with HIV acquired through male-to-male sexual contact exhibited the highest suppression rates, rising from 66.10% in 2017 to 67.90% in 2022. This group consistently showed the highest levels of viral suppression throughout the period. Conversely, those with HIV from injection drug use had the lowest suppression rates, starting at 54.90% and decreasing slightly to 54.80% by 2022. Similarly, a marginal increase in viral suppression was reported in individuals with HIV through both male-to-male sexual contact and injection drug use from a rate of 63.50% in 2017 to 64.90% in 2022. For heterosexual contact, viral suppression rates improved from 60.90% in 2017 to 63.20% in 2022. Individuals with HIV acquired through other modes of transmission showed an increase from 52.90% to 56.90% over the same period.

Racial and ethnic disparities in viral suppression rates among people with HIV

**Figure 4:**
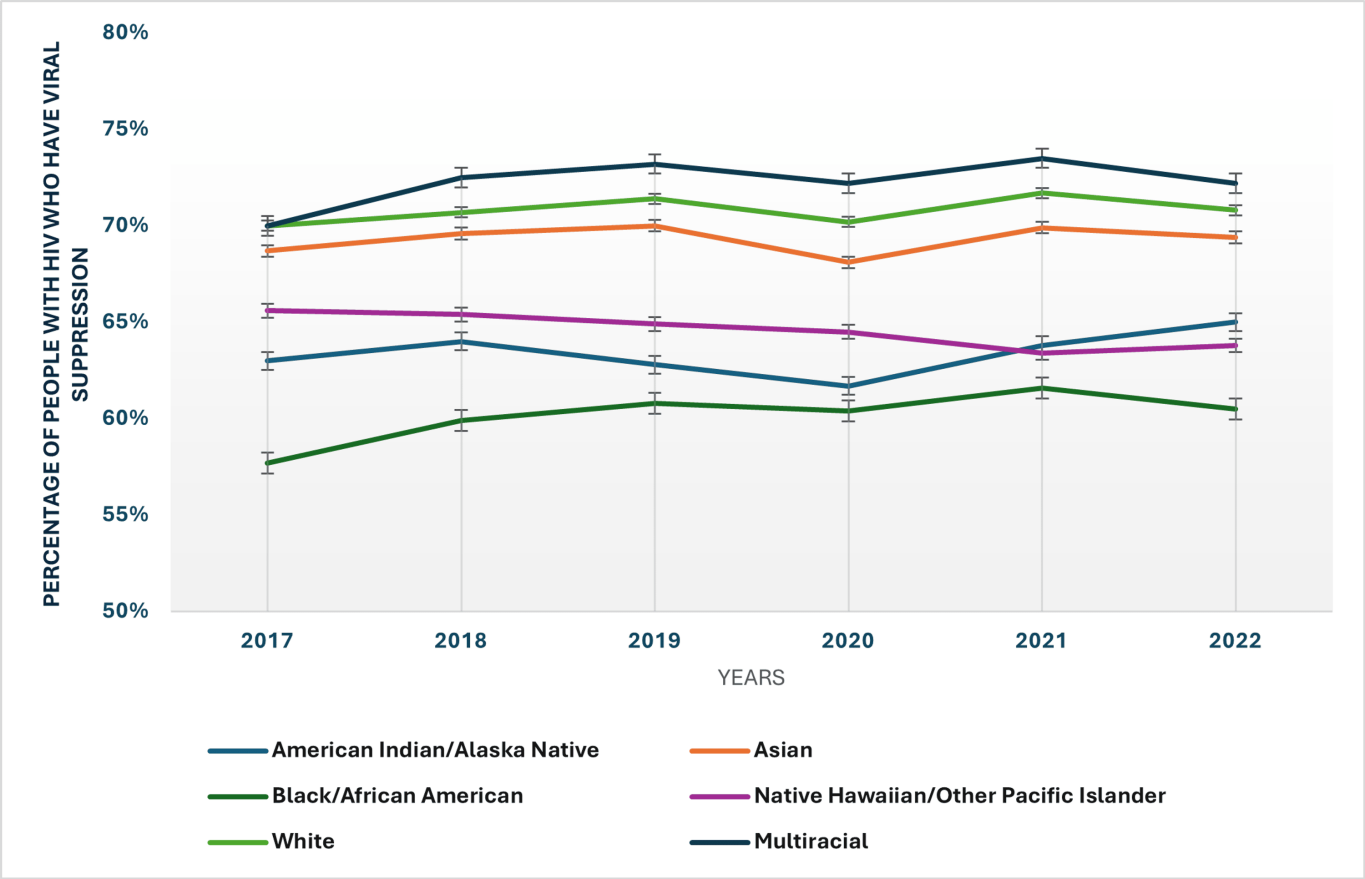
Racial and ethnic disparities in viral suppression rates HIV: Human immunodeficiency virus

From 2017 to 2022, viral suppression rates among people with HIV varied significantly across racial and ethnic groups (Figure 4). White individuals exhibited the lowest suppression rates, starting at 70% in 2017 and reaching 70.80% by 2022. On the other hand, multiracial individuals showed a high suppression rate, starting from 70% in 2017 and increasing to 72.20% in 2022. The Asian individuals also demonstrated relatively high rates, starting at 68.70% in 2017 and reaching a suppression rate of 69.40% in 2022. The Black/African American population presented the highest suppression rates among the reported groups, beginning at 57.70% in 2017 and increasing to 60.50% in 2022. Still, American Indian/Alaska Native individuals had a higher suppression rate that increased from 63% in 2017 to 65% in 2022, similar to the Hispanic/Latino individuals who showed significant improvement, with rates rising from 62.50% in 2017 to 64.30% in 2022. Native Hawaiian/Other Pacific Islander rates fluctuated significantly, from 65% in 2017 to 63.80% in 2022.

## Discussion

This study provides a comprehensive analysis of disparities in HIV PrEP utilization and viral suppression rates, shedding light on areas of progress and persistent challenges. The findings align with and extend previous research, offering insights into how demographic factors influence PrEP uptake and viral suppression outcomes. Moreover, the marked increase in PrEP prescriptions from 13.20% to 36% of those eligible, alongside the rise in the number of prescriptions from 161,185 to 437,425, is a positive development. This trend reflects broader efforts to expand access to HIV prevention tools and is consistent with previous studies documenting increased PrEP uptake due to enhanced awareness and accessibility [11-14]. For instance, Sullivan et al. reported a substantial rise in PrEP use from 3.3/100,000 in 2012 to 36.7 in 2017, with an annual growth rate of 56% [11]. Similarly, targeted public health campaigns and expanded insurance coverage have driven this increase [12-14]. Despite this progress, PrEP uptake remains suboptimal among certain populations, underscoring ongoing barriers.

Our analysis reveals significant variations in PrEP utilization and viral suppression across age groups, mirroring earlier studies. Individuals aged 13-24 demonstrated notably lower PrEP uptake than older groups. This observation supports previous research highlighting barriers younger populations face, including stigma, lack of awareness, and limited access to healthcare services [11,15]. While PrEP prescriptions have increased in this age group over time, uptake remains lower than in older demographics, indicating persistent challenges in reaching younger individuals with effective prevention strategies [15]. Conversely, older age groups, particularly those aged 25-34 and 35-44, exhibited higher PrEP utilization and viral suppression rates. This finding aligns with Sullivan et al.'s report of increased PrEP use among 25-34-year-olds (estimated annual percentage change (EAPC): +61%) compared to older groups (EAPC: +52%) [11]. The higher PrEP uptake in these groups likely reflects better healthcare access and a heightened perceived risk of HIV, leading to more proactive engagement in preventive measures [16]. The lack of data for individuals aged 55-64 in earlier years presents a limitation that future research should address.

Gender disparities in PrEP utilization and viral suppression are evident in our study. Men, especially those who have sex with men, showed higher PrEP prescription rates compared to women. This disparity aligns with previous studies, which reported higher growth rates of PrEP use among men (EAPC: +68%) compared to women (EAPC: +5%) [8,9,11]. Lower PrEP utilization among women may be linked to gender-specific barriers, including access issues, lower perceived risk of HIV, and social stigma [14,17]. Transgender individuals also showed varied PrEP utilization rates, with transgender men experiencing significant increases over time. This trend is consistent with Ogunbajo et al.'s findings of growing awareness and access to PrEP among transgender populations [18]. However, the limited data on transgender individuals and additional gender identities highlights a gap in research, indicating a need for more inclusive data collection. Racial and ethnic disparities in PrEP utilization and viral suppression were pronounced in our analysis. White individuals consistently showed higher PrEP utilization rates compared to Black/African American and Hispanic/Latino individuals. This observation aligns with previous research identifying racial disparities in PrEP access and adherence [19,20]. Sullivan et al. reported that despite overall increases, PrEP use was inequitable by race, ethnicity, and sex, with the highest rates among White individuals and the lowest among Black individuals [19]. Lower PrEP uptake among Black/African American and Hispanic/Latino populations likely reflects systemic barriers, including socioeconomic challenges, limited healthcare access, and cultural factors influencing health behaviors [20]. The absence of data for certain racial and ethnic groups, such as American Indian/Alaska Native and Asian populations, underscores the need for more comprehensive data collection to understand better and address disparities.

The modest increase in viral suppression rates from 63.10% to 65.10% suggests incremental progress in treatment outcomes. Previous research has also noted gradual improvements in viral suppression rates attributed to advancements in antiretroviral therapies and improved adherence strategies [21]. However, the slower rate of increase in viral suppression compared to PrEP utilization may reflect challenges in treatment adherence and access to care. Earlier studies have highlighted that achieving and maintaining viral suppression can be impeded by factors such as healthcare access, socioeconomic status, and stigma [22]. For example, the higher viral suppression rates among White individuals compared to Black/African American and Hispanic/Latino individuals reflect disparities in healthcare access and treatment adherence. This finding is consistent with previous research indicating that racial and ethnic minorities face barriers to achieving optimal HIV treatment outcomes [23]. Addressing these disparities requires targeted interventions to improve access to care and support adherence among underserved populations. While the increase in PrEP utilization is promising, significant disparities persist across age, gender, race, and ethnicity. The study highlights the need for continued efforts to enhance PrEP access and support for marginalized groups, ensuring that all individuals at risk of HIV have equitable access to prevention and treatment resources.

Strengths and limitations of this study

This study utilizes the AHEAD National Database, a comprehensive tool that provides extensive data on HIV-related indicators across various demographics. The analysis includes a broad range of variables, including age, gender, and race/ethnicity, allowing for an in-depth examination of disparities in PrEP uptake and viral suppression. The large sample size and longitudinal data enhance the reliability of trends over time. The study is constrained by data availability, particularly for certain racial and ethnic groups, which limits the ability to assess disparities fully. Additionally, while the database offers valuable insights, it may not capture all PrEP utilization and viral suppression nuances, such as socio-economic factors or regional differences. The lack of data for some variables in earlier years further complicates trend analysis and interpretation.

Implications of the findings for public health and policy

Although PrEP use has significantly increased between 2017 and 2022, particularly in younger persons, there is still a need for the implementation of targeted public health interventions as a means of increasing awareness of the importance of PrEP in HIV prevention among different groups. Racially and demographically appropriate public health interventions are recommended to ensure that the different needs and hindrances experienced by different groups are aptly tackled as a means of increasing PrEP use. For instance, interventions that include consideration of alternative PrEP provision strategies that include telemedicine delivery of PrEP and pharmacy-based PrEP deliveries, carrying out gain-based stigma-elimination campaigns, and enhancing the capacity for reimbursement for the PrEP medications are recommended. Moreover, structural barriers affecting the access to PrEP medications by different racial and demographic groups, including insurance coverage, costs, and availability of services should be addressed. Implementation of these recommendations is important and relevant to the United States, given that they will not only increase access to HIV medications and treatment, but will additionally address the existing racial and demographic disparities observed with regard to access to important medications, including PrEP, and treatment. Moreover, implementing the recommended public health interventions aimed at increasing awareness and uptake of PrEP medications will aid in the reduction of HIV infection rates in the United States.

## Conclusions

This study highlights significant disparities in PrEP utilization and viral suppression across age, gender, race, and ethnicity. While there has been progress in increasing PrEP uptake and achieving viral suppression, these disparities indicate a need for targeted interventions and continued efforts to ensure equitable access to HIV prevention and care. Addressing these disparities is essential for achieving the goals of the Ending the HIV Epidemic initiative and improving health outcomes for all individuals at risk for or living with HIV. The alignment of these findings with previous research underscores the importance of ongoing efforts to address health disparities and promote equitable HIV care.

## References

[1] Glenshaw MT, Gaist P, Wilson A, Cregg RC, Holtz TH, Goodenow MM (2022). Role of NIH in the ending the HIV epidemic in the US initiative: research improving practice. J Acquir Immune Defic Syndr.

[2] (2024). HIV declines among young people and drives overall decrease in new HIV infections.. https://www.cdc.gov/media/releases/2023/p0523-hiv-declines-among-young-people.html.

[3] (2021). The estimated lifetime medical cost of chlamydia, gonorrhea, and trichomoniasis in the United States, 2018: erratum. Sex Transm Dis.

[4] Remien RH, Stirratt MJ, Nguyen N, Robbins RN, Pala AN, Mellins CA (2019). Mental health and HIV/AIDS: the need for an integrated response. AIDS.

[5] Hamilton DT, Hoover KW, Smith DK (2023). Achieving the "ending the HIV epidemic in the U.S." incidence reduction goals among at-risk populations in the South. BMC Public Health.

[6] Agrahari V, Anderson SM, Peet MM, Wong AP, Singh ON, Doncel GF, Clark MR (2022). Long-acting HIV pre-exposure prophylaxis (PrEP) approaches: recent advances, emerging technologies, and development challenges. Expert Opin Drug Deliv.

[7] Le Guillou A, Buchbinder S, Scott H, Liu A, Havlir D, Scheer S, Jenness SM (2021). Population impact and efficiency of improvements to HIV PrEP under conditions of high ART coverage among San Francisco men who have sex with men. J Acquir Immune Defic Syndr.

[8] Sepodes B, Rocha J, Batista J, Figueira ME, Dráfi F, Torre C (2021). Implementation and access to pre-exposure prophylaxis for Human immunodeficiency virus by men who have sex with men in Europe. Front Med (Lausanne).

[9] Odii IO, Vance DE, Patrician PA (2024). HIV PrEP coverage among black adults: a concept analysis of the inequities, disparities, and implications. Health Equity.

[10] Sullivan PS, Woodyatt CR, Kouzouian O, Parrish KJ, Taussig J, Conlan C, Phillips H (2022). America's HIV epidemic analysis dashboard: protocol for a data resource to support ending the HIV epidemic in the United States. JMIR Public Health Surveill.

[11] Sullivan PS, Giler RM, Mouhanna F (2018). Trends in the use of oral emtricitabine/tenofovir disoproxil fumarate for pre-exposure prophylaxis against HIV infection, United States, 2012-2017. Ann Epidemiol.

[12] Garrison LE, Haberer JE (2021). Pre-exposure prophylaxis uptake, adherence, and persistence: a narrative review of interventions in the U.S. Am J Prev Med.

[13] MacDonald J, Estcourt CS, Flowers P (2023). Improving HIV pre-exposure prophylaxis (PrEP) adherence and retention in care: process evaluation and recommendation development from a nationally implemented PrEP programme. PLoS One.

[14] Calabrese SK, Dovidio JF, Tekeste M (2018). HIV pre-exposure prophylaxis stigma as a multidimensional barrier to uptake among women who attend planned parenthood. J Acquir Immune Defic Syndr.

[15] Jaiswal J, Griffin M, Singer SN (2018). Structural barriers to pre-exposure prophylaxis use among young sexual minority men: the P18 cohort study. Curr HIV Res.

[16] Pleuhs B, Quinn KG, Walsh JL, Petroll AE, John SA (2020). Health care provider barriers to HIV pre-exposure prophylaxis in the United States: a systematic review. AIDS Patient Care STDS.

[17] Aidoo-Frimpong G, Orom H, Agbemenu K, Collins RL, Morse GD, Nelson LE (2022). Exploring awareness, perceptions, and willingness to use HIV pre-exposure prophylaxis: a qualitative study of Ghanaian immigrants in the United States. AIDS Patient Care STDS.

[18] Ogunbajo A, Storholm ED, Ober AJ (2021). Multilevel barriers to HIV PrEP uptake and adherence among black and Hispanic/Latinx transgender women in Southern California. AIDS Behav.

[19] Sullivan PS, DuBose SN, Castel AD (2024). Equity of PrEP uptake by race, ethnicity, sex and region in the United States in the first decade of PrEP: a population-based analysis. Lancet Reg Health Am.

[20] Mayer KH, Agwu A, Malebranche D (2020). Barriers to the wider use of pre-exposure prophylaxis in the United States: a narrative review. Adv Ther.

[21] Li MJ, Su E, Garland WH (2020). Trajectories of viral suppression in people living with HIV receiving coordinated care: differences by comorbidities. J Acquir Immune Defic Syndr.

[22] Billock RM, Samoff E, Lund JL, Pence BW, Powers KA (2021). HIV viral suppression and pre-exposure prophylaxis in HIV and syphilis contact tracing networks: an analysis of disease surveillance and prescription claims data. J Acquir Immune Defic Syndr.

[23] Blank AE, Fletcher J, Verdecias N, Garcia I, Blackstock O, Cunningham C (2015). Factors associated with retention and viral suppression among a cohort of HIV+ women of color. AIDS Patient Care STDS.

